# The Role of Vitamin D3 in Ocular Diseases

**DOI:** 10.3390/nu16121878

**Published:** 2024-06-14

**Authors:** Małgorzata Mrugacz, Kamila Pieńczykowska, Anna Bryl

**Affiliations:** 1 Department of Ophthalmology and Eye Rehabilitation, Medical University of Bialystok, 15-089 Białystok, Poland; annabryl@interia.pl; 2 The Medical University of Bialystok Clinical Hospital, 15-089 Białystok, Poland; mynameiskama@gmail.com

**Keywords:** vitamin D, eye disease, cholecalciferol, glaucoma, keratoconus

## Abstract

Vitamin D3 plays a vital role in numerous physiological processes within the human body, including having a positive effect on eye health. It is renowned for its immunomodulatory, anti-inflammatory, antioxidant, and angiogenic properties. Its deficiency is evolving into a significant global challenge. In order to explain the connection between vitamin D3 and various ocular diseases, 84 relevant studies, mainly from the PubMed database, published in English between 1999 and 2024 were analyzed. Ocular tissues can activate and regulate vitamin D levels, which emphasizes the significance of this nutrient in maintaining eye homeostasis. While there is suggestive evidence for a probable association between vitamin D3 and ocular health, more robust research is needed to establish causation and inform clinical guidelines.

## 1. Introduction

Vitamin D_3_, also known as cholecalciferol, is one of the key precursors of vitamin D, next to vitamin D_2_ or ergocalciferol. Vitamin D_3_ is primarily known for its involvement in calcium homeostasis and bone metabolism; it also exhibits immunomodulatory, anti-inflammatory, antioxidant, and angiogenic properties. Vitamin D_3_ is generated from its precursor, 7-dehydrocholesterol, in the epidermal layer of the skin during exposure to sunlight. After undergoing metabolic processes in the liver and kidneys, vit. D_3_ is converted into its biologically active forms, namely 25-hydroxyvitamin D (25(OH)D_3_) in the liver and 1,25-dihydroxyvitamin D (1,25(OH)_2_D_3_) in the kidneys. According to the stance of the Endocrine Society, the optimal range for serum vitamin D levels is between 40 and 60 ng/mL. To maintain this range, the daily intake should be 400 to 1000 International Units (IU) for infants under one year, 600 to 1000 IU for children and adolescents aged 1 to 18 years, and 1500 to 2000 IU for adults. Deficiency is classified as a level below 30 ng/mL, while insufficiency falls within the range of 20 to 30 ng/mL [1]. High prevalence rates of vitamin D_3_ deficiency are reported across Europe, with northern countries experiencing a more significant deficiency due to limited sunlight during long winters. Studies show that around 40% of the European population is deficient. In the United States and Canada, vitamin D_3_ deficiency affects about 24–42% of the population. In countries such as India, Pakistan, and Bangladesh the prevalence ranges from 70% to 90%. The groups who are at risk of developing vitamin D deficiency are elderly people (reduced skin capacity to synthesize vitamin D_3_ and limited outdoor activities increase their risk), dark-skinned individuals (higher melanin levels reduce the skin’s ability to produce vitamin D_3_), infants and children (inadequate dietary intake and less sun exposure contribute to higher deficiency rates), pregnant and lactating women (increased demand for vitamin D_3_ can lead to deficiencies affecting both mothers and infants), and individuals with limited sun exposure (those living at high latitudes, working indoors, or adhering to strict clothing customs are at greater risk). The typical method for assessing an individual’s vitamin D levels is through the measurement of serum 25(OH)D_3_.

The aim of this review is to summarize and update the existing level of knowledge about vitamin D in eye diseases by taking into account the latest articles found in the PubMed database (from 1999 to 2024) on similar topics.

## 2. Method

**Figure 1:**
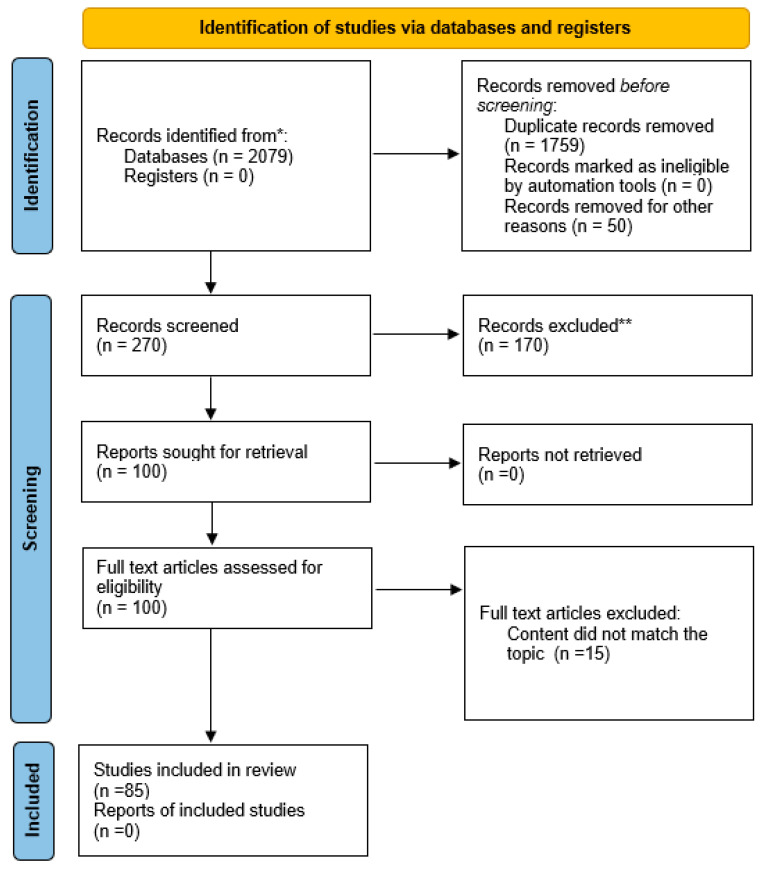
The PRISMA flowchart. * Consider, if feasible to do so, reporting the number of records identified from each database or register searched (rather than the total number across all databases/registers). ** If automation tools were used, indicate how many records were excluded by a human and how many were excluded by automation tools.

This systematic review was carried out and documented in accordance with the guidelines set forth by the PRISMA statement for systematic reviews and meta-analyses, as well as the PRISMA extension statement for network meta-analyses [2]. The workflow for the identification and stepwise selection of the studies is presented in Figure 1. The Systematic Review Registration: PROSPERO registration no 555572.

It was based mainly on the PubMed online database, with a focus on articles published in English between 1999 and 2024. We used the following key terms: “vitamin D” or “cholecalciferol” in combination with “ocular”, “eye disease”, “glaucoma”, “dry eye”, “myopia”, “keratoconus”, “thyroid eye disease”, “retinoblastoma”, “diabetic retinopathy”, “age-related macular degeneration”, “cataract”. We used all types of publications. Studies were disqualified if they were duplicates, conference abstracts or posters, or not relevant to the research question. Finally, we chose 84 articles to include in this review: 8 related to dry eye disease, 7—myopia, 8—keratoconus, 8—Graves’ orbitopathy, 6—retinoblastoma, 12—age-related macular degeneration, 14—diabetic retinopathy and diabetic macular edema, 12—glaucoma, 7—cataract.

## 3. Dry Eye Disease (DED)

Low levels of vitamin D can contribute to dry eye disease, a common ocular condition which manifests itself as dryness, discomfort, redness and irritation of the eyes. Patients diagnosed with DED turned out to have decreased vitamin D_3_ levels in comparison to those without dry eyes. There was no preference based on gender or alteration in prevalence with advancing age [3]. However, another study indicated differences in the impact of vitamin D based on gender: the tear break-up time test (TBUT) showed improvement in both male and female patients, while the fluorescein staining score (FSS) and tear secretion exhibited enhancement specifically in women [4]. Inflammation is recognized as a key mechanism in dry eye disease. The anti-inflammatory effects of activated vitamin D involve inhibiting the activation of T-helper cells and cytotoxic T cells, as well as reducing the production of inflammatory mediators such as interleukins IL-2, IL-6, IL-8, and IL-12. Additionally, vitamin D suppresses inflammatory agents such as C-reactive protein (CRP), tumor necrosis factor (TNF)-α, IL-1, and IL-6, while promoting the production of IL-10 [5,6]. Insufficient levels of vitamin D can contribute to the dry eye syndrome, which can cause conjunctival squamous metaplasia and the reduction of goblet cells on the eye’s surface [7]. The vitamin D levels among individuals diagnosed with dry eye did not demonstrate any correlation with the Ocular Surface Diseases Index (OSDI) scores or the IL-6 levels. However, the IL-6 levels exhibited a correlation with tear production [8]. A lack of vitamin D is linked to more severe subjective symptoms and reduced tear production in individuals suffering from dry eye [9]. By adding vitamin D supplementation to conventional dry eye therapy, the stability and osmolarity of tears can be enhanced [10]. Nevertheless, there was no consistent correlation between vitamin D_3_ deficiency and the severity of DED.

## 4. Myopia

Myopia, commonly known as nearsightedness, is a refractive error of the eye, where close objects appear clearly while distant objects appear blurry. This occurs when the eyeball is too long or the cornea (the clear front cover of the eye) is too curved, causing light rays to focus in front of the retina instead of directly on it. Individuals who lacked sufficient levels of vitamin D showed a notably higher occurrence of myopia compared to those with adequate levels [11]. An analysis performed in a group of patients aged between 12 and 50 years old indicated that the people who suffered from myopia had lower serum vit. D levels than the healthy ones. The mechanisms involved in this relation in human beings remain unexplained [12]. One of the most recent studies, which aimed to explore the function of vitamin D_3_ in myopia development, was carried out on mice. Calcipotriol (a vitamin D_3_ analogue) apparently triggers a signaling pathway dependent on the scleral vitamin D receptor, elevating the levels of α1 chain of type I collagen expression in the sclera [13]. Regarding younger children (aged 5–15 years), there appears to be no remarkable connection between vitamin D deficiency and myopia [14]. A Chinese cross-sectional study carried out in a similar age group (6–14 years old) also concluded that there was no association between the serum 25(OH)D concentration and myopia [15]. Nevertheless, an Indian study assessing children aged 5–15 years presented an opposing outcome: vitamin D_3_ was suggested to play a pivotal role in the onset of myopia among children [16]. Greater exposure to UVB radiation was linked to a decrease in myopia, especially during the adolescent and young adult stages [17].

## 5. Keratoconus (KC)

In keratoconus, the cornea thins and gradually bulges outward, forming a cone-like shape. People suffering from this disease have the tendency to have lower serum levels of 25-hydroxyvitamin D than healthy ones [18]. Elevated levels of inflammatory mediators and immune elements have been noted in the cornea, tear fluid, and bloodstream of individuals suffering from this ailment, while anti-inflammatory agents such as vitamin D and their receptors were diminished [19]. The presence of vitamin D receptor polymorphism has been linked to the onset of keratoconus. Specifically, the Taq1 gene and its tt alleles have been identified as significant risk factors for the development of this condition [20]. Collagen, a protein that provides strength and structure to various tissues in the body, including the cornea, plays a crucial role in the development of keratoconus. A correlation between vit. D deficiency and higher levels of systemic biomarkers of collagen degradation has been scientifically proven. A low level of 25(OH)D plays a part not only in the onset of KC, but also in its progression. This applies to both progressive and nonprogressive KC groups of patients [21,22]. Considering its occurrence pattern and the improved response to increased vitamin D intake, this condition could be likened to an eye-related form of “vitamin D-resistant rickets”. This explanation also clarifies why keratoconus may recur after corneal grafting, as patients likely maintain or worsen their vitamin D deficiency as they age [23]. Thus, supplementation of vitamin D can become an alternative way of treatment to surgery, as it provides the stabilization of KC progression after 12 months [24]. The meta-analysis by Gupta PC suggested that a low level of vitamin D can be associated with higher odds of severe keratoconus. Thus, routine monitoring of vitamin D levels and trace elements in KC patients at the time of diagnosis and during subsequent follow-up appointments could serve as a predictive measure for assessing disease severity [25].

## 6. Graves’ Orbitopathy

Graves’ orbitopathy or thyroid eye disease (TED) is a fundamental extrathyroidal manifestation of Graves’ disease (GD). It may be revealed by lid retraction, protrusion of the eye, eye’s soft tissue involvement, spontaneous retrobulbar pain, and discomfort on an attempted upward or downward gaze. People with GD tend to have lower vitamin D levels as compared to the general population [26]. Patients with thyroid eye disease exhibit significantly lower serum 25(OH)D levels compared to those with Graves’ disease (24.8 ± 13.2 ng/mL vs. 29.4 ± 13.3 ng/mL; *p* = 0.006) [27]. As shown in a case report from Saudi Arabia, a lack of vitamin D might worsen the start or progression of Graves’ disease, but addressing the deficiency could potentially reverse it. The cause of this phenomenon might be the connection between the vitamin D-receptor gene and the vitamin D-binding protein gene polymorphisms with Graves’ disease [28]. Thyrotoxicosis adversely affects bone health and exhibits intricate dynamics in bone and vitamin D metabolism [29]. Another important characteristic of vitamin D_3_ is its effectiveness in mitigating glucocorticoid side effects when it comes to the treatment of Graves’ orbitopathy. Patients receiving methylprednisolone therapy who had low vitamin D levels or bone mass were given calcium and vitamin D supplements, along with encouragement to increase sunlight exposure. This likely contributed to the higher vitamin D levels observed after treatment [30]. The latest publications showed no significant connection between the prevalence of vitamin D deficiency and TED, mainly in terms of patients with 25(OH)D > 20 ng/mL [31,32]. A different study by G. Lanzolla, conducted on a relatively large population, found no differences in 25-hydroxyvitamin D levels between patients with and without Graves’ orbitopathy (GO). Additionally, no associations between serum 25-hydroxyvitamin D levels and GO characteristics were identified, except for an inverse correlation with eyelid aperture [33]. Further research is needed to investigate the potential influence of a severe vitamin deficiency (less than 10 ng/mL) on the likelihood of developing Graves’ ophthalmopathy. Well-designed clinical trials are vital to evaluate the efficacy and safety of vitamin D_3_ supplementation as an adjunctive therapy in Graves’ orbitopathy management.

## 7. Retinoblastoma (RB)

Retinoblastoma is the second most common intraocular malignant tumor after uveal melanoma [34]. A study investigating the influence of nutrients taken by mothers during pregnancy on the development of sporadic unilateral RB in their offspring concluded that the enhancement of the serum vitamin D level lowers the probability of occurrence of this condition [35]. Another research conducted on mice demonstrated an analogue of vitamin D_3_-1,25-dihydroxy-16-ene-23-yne-vitamin D_3_ (16,23-D_3_) as being particularly worthy of attention. Intraperitoneal 16,23-D_3_ injections notably hindered the development of Y-79 human retinoblastoma cells under the skin of athymic nude mice [36]. In the athymic mice involved in the large-tumor experiment, both 1α-OH-D_2_ and 16,23-D_3_ effectively suppressed tumor growth in comparison to the control group. However, in the extended study, while 1α-OH-D_2_ restrained tumor growth, 16,23-D_3_ did not exhibit the same inhibitory effect [37]. Calcitriol blocks the proliferation of smooth muscle cells by halting cell cycle progression rather than triggering apoptosis. The specific signaling pathways through which vitamin D_3_ impeded cell growth are yet to be fully understood, but they included reducing the hyperphosphorylation of the Rb protein and Chk1 phosphorylation, without affecting other cell cycle inhibitors [38]. The inclusion of vitamin D proves to be beneficial during chemotherapy for RB with cisplatin. This combination might enable a reduction in the amount of cisplatin needed, potentially lowering both short-term and long-term toxicity while still upholding its potent anti-cancer properties [39]. Despite these promising theoretical benefits, direct evidence linking vitamin D_3_ specifically to retinoblastoma is limited, and more research is needed to understand this relationship fully. Future research directions could include clinical trials to evaluate the potential benefits of vitamin D_3_ supplementation in preventing retinoblastoma or as an adjunctive therapy in treating the disease. Studies using animal models of retinoblastoma to assess the effects of vitamin D_3_ on tumor growth, progression, and response to treatment are also needed.

## 8. Age-Related Macular Degeneration (AMD)

Age-related macular degeneration leads to blur or complete loss of central vision. Individuals who suffer from this condition tend to have lower levels of vitamin D than healthy ones [40,41,42]. Vitamin D_3_ exhibits protective properties against oxidative stress. It enhances cell viability, diminishes apoptosis, reduces DNA damage markedly across all concentrations between 10 and 100 nM, lowers intracellular reactive oxygen species generation notably within the range of 10–60 nM, and elevates mitochondrial membrane potential significantly within concentrations ranging from 10 to 50 nM in the cells of retinal pigment epithelium [43]. An elevated level of serum 25(OH)D was associated with a higher likelihood of early AMD in a group of patients aged 60 years or younger. However, among individuals older than 60, it was linked to a reduced risk of late AMD. The same study suggests that maintaining serum 25(OH)D within certain levels may have benefits in reducing the risk of AMD for at-risk individuals [44]. There seems to be a tendency for late-stage AMD in humans whose serum vitamin D levels are below 50 nmol/L [45]. The level of vitamin D_3_ might affect the occurrence of neovascular AMD, implying that, as the levels of 25(OH)D_3_ decrease, the frequency of AMD may increase [46]. The combination of vitamin D_3_ and meso-zeaxanthin provided protection for the retinal pigmented epithelium against multiple damages that resemble the complex pathogenic processes seen in AMD [47]. According to the study of D’Aloisio R, oral treatment with vitamin D_3_ might cause a significant increase in choroidal thickness and choriocapillary vessel density [48]. The combination of a nutritional antioxidant complex and vitamin D appears as a more efficient oral supplementation treatment for the early stage of AMD [49]. Consuming a diet abundant in vitamin D could potentially hinder or postpone the advancement to severe stages of age-related macular degeneration [50,51]. More research is needed in order to establish clinical guidelines regarding the optimal vitamin D_3_ levels for AMD prevention or management.

## 9. Diabetic Retinopathy (DR) and Diabetic Macular Edema (DME)

An abnormal production of reactive oxygen species and chronic inflammation are credited as significant factors contributing to the development of diabetic retinopathy (DR) [52]. A low cholecalciferol serum concentration heightens the risk of acquiring DR in people suffering from type 2 diabetes mellitus [53]. The indicators of inadequate vitamin D levels are also linked to the severity of DR [54]. Insufficient levels of vitamin D could hasten the onset of diabetic retinopathy [55]. Vitamin D deficiency and the probability of developing proliferative retinopathy are closely linked due to its capacity to hinder the formation of new blood vessels (neovascularization). Patients whose serum vit. D levels reached 20 mg/dL or less became more prone to come down with proliferative DR in comparison to those with non-proliferative DR or without retinopathy [56]. Vitamin D-binding protein (VDBP) levels are reduced in diabetic individuals with retinopathy, potentially due to VDBP being excreted in the urine because of tubular dysfunction in diabetic patients. Alternatively, decreased VDBP levels could contribute to the advancement of DR by obstructing the microvessels in the retina [57]. Regular vit. D consumption reduces random and fasting blood glucose levels by increasing retinoblastoma protein concentration. Vitamin D also influences insulin sensitivity by either boosting the number of insulin receptors or enhancing the responsiveness of insulin receptors to insulin [58]. Treatment with vitamin D_3_ successfully repaired the compromised integrity of the blood–retinal barrier, which had been significantly affected by exposure to high glucose levels [59]. Vitamin D_3_ has the potential to safeguard the typical structure of the retina, ease retinal vascular permeability, and suppress the apoptosis of retinal cells. This process is possible thanks to the vitamin’s ability to reduce the reactive oxygen species resulting from diabetes by inhibiting the ROS/TXNIP/NLRP3 pathway [60]. Supplementing with vitamin D could potentially be important in preventing, treating, and enhancing the prognosis of proliferative diabetic retinopathy because it can also hinder the vascular endothelial growth factor (VEGF) [61]. Moreover, the combination of curcumin and homotaurine with vitamin D_3_ has demonstrated effectiveness in altering the mean concentrations of inflammatory and retinal damage mediators [62]. Thus, cholecalciferol presents itself as beneficial in diabetes control and the prevention of DR development. To the contrary, the Mendelian randomization study revealed no notable causal link between serum 25(OH)D levels and DR [63]. According to Seyyar SA, people with diabetes tend to have lower levels of 25-hydroxyvitamin D compared to those without diabetes, but there is not a straightforward connection between 25-hydroxyvitamin D levels and the occurrence of diabetic retinopathy [64].

Diabetic macular edema may manifest at different levels of DR, encompassing mild, moderate, and severe non-proliferative DR (NPDR), as well as proliferative DR (PDR). This condition is identified by the leakage of retinal vessels, compromised endothelial integrity, and the accumulation of exudative fluid in the macula. The prevalence of DME corresponds with inadequate vitamin D concentrations [42]. Other results indicate that rectifying vitamin D deficiency (VDD) in DME patients receiving anti-VEGF therapies has a positive impact on sustaining the improvement in visual acuity and reducing central macular thickness observed with these treatments over a period of six months [65].

## 10. Glaucoma

At present, glaucoma is a leading reason of irreversible blindness worldwide. Increased intraocular pressure (IOP) is a widely known determinant, although not necessary for the advance of glaucoma [66]. Vitamin D_3_ supplementation does not alter the IOP of healthy people [67]. Insufficient vitamin D levels, along with the presence of the BsmI ‘B’ allele and the TaqI ‘t’ allele, are notable risk factors implicated in the onset of glaucoma [68]. Calcitriol safeguards the retinal ganglion cells, maintaining retinal function, diminishing inflammatory cytokines, and boosting the expression of neuroprotective factors in glaucomatous neurodegeneration [69]. 25-(OH)_2_D_3_ counteracted various H_2_O_2_-induced changes in extracellular matrix (ECM) proteins associated with glaucomatous effects on cellular activity, proliferation, stress response, and ECM synthesis in human trabecular meshwork cells. Specifically, calcitriol inhibited the synthesis of matrix metalloproteinases and tissue inhibitors of metalloproteinases, fibroblast proliferation, collagen synthesis, apoptosis of HTMCs, and inflammation by inhibiting interleukins. Additionally, it reversed the up-regulation of transforming growth factor-beta (TGF-β) and SMAD3 protein, and the down-regulation of the vitamin D receptor (VDR) induced by oxidative stress in HTMCs [70]. One of the most recent studies investigating the relationship between serum vitamin D levels and ocular hypertension among the Korean population proved their inverse dose-dependent correlation—people who suffered from vitamin D deficiency were more likely to present elevated intraocular pressure. This finding was especially relevant in male and young age groups, indicating a possible correlation between vitamin D and sex hormones, such as estrogen, follicular stimulating hormone, or anti-Müllerian hormone. Further research and clinical trials are recommended to ascertain this dependency [71]. On the other hand, a different study, exploring the impact of 1,25-dihydroxycholecalciferol deficiency on the risk of high IOP in a group of patients with chronic illnesses, concluded that females are more affected. Moreover, the findings suggest that vitamin D status independently influences the pathophysiology of glaucoma, particularly as a secondary aggravating factor. In the presence of a primary factor, havinglow levels of vitamin D may increase the susceptibility of the optic nerve or its surroundings to damage from glaucoma [72]. Taking into consideration the progression of glaucoma, there seems to be no connection with serum vitamin D levels. If vitamin D is expected to provide metabolic protection against the advancement of glaucoma, there is no apparent clinical manifestation of these changes [73]. A similar conclusion was drawn in a pilot study from Saudi Arabia, which aimed to investigate the association of decreased serum 25-OHD concentration with the cup/disc (C/D) ratio in primary open-(POAG) and closed-angle glaucoma (PACG). Despite low serum vitamin D levels in both the glaucomatous and control subjects, the study concluded that there was no association between vitamin D deficiency and the progression of POAG and PACG in the Saudi population [74]. Therefore, reduced levels of serum 25-OHD were linked to the existence of POAG, but did not show an association with its severity. Further longitudinal studies are suggested to explore the potential impact of vitamin D supplementation on glaucoma progression. Vitamin D_3_ seems capable of inhibiting the proliferation, migration, and transdifferentiation of human Tenon’s fibroblast cells, which is crucial in optimizing the outcome of trabeculectomy by maintaining aqueous filtration [75]. The levels of vitamin D found in the aqueous humor were notably lower in patients with both cataracts and glaucoma compared to those with cataracts alone, which means vit. D is a promising candidate in glaucoma treatment [76]. In terms of glaucoma treatment and its prevention, the combination of vitamin D_3_, gastrodin, vitamin C, blackcurrant, and lycopene needs to be mentioned. Research indicates a significant capability to reverse damage induced by glaucoma, by reducing the generation of reactive oxygen species and promoting cell survival through the suppression of apoptosis. These effects were corroborated by the activation of an intracellular mechanism following the administration of the compound, whether administered before or after inducing glaucoma [77]. Despite the intriguing findings, the clinical implications of vitamin D supplementation in glaucoma management are not yet well-established. There is a need for more solid, randomized controlled trials to determine whether vitamin D_3_ supplementation can be effectively used as a preventive or therapeutic measure for glaucoma. Moreover, studies focusing on diverse populations would be beneficial to understand how genetic, environmental, and lifestyle factors might influence the relationship between vitamin D_3_ and glaucoma.

## 11. Cataract

Cataract refers to the clouding of the lens located within the eye, resulting in a loss of transparency. While it can occur at any stage of life, it is predominantly observed in older individuals. Rahman ST discovered that regularly providing high-dose vitamin D supplements to elderly individuals residing in regions where vitamin D deficiency is uncommon is improbable to decrease the necessity for cataract surgery [78]. However, the study by Abdellah MM discovered that patients with cataract are prone to having a severe deficiency of vit. D [79]. The study by Liu H confirmed the substantial causal association between vitamin D deficiency and cataract risk by substituting the vitamin D measurement tools used in European populations with those used in Japanese populations [80]. A deficiency in vitamin D was linked to posterior subcapsular (PSC) cataracts, indicating that increasing vitamin D intake may lower the occurrence of PSC cataracts. A lack of vitamin D influences the calcium metabolism of lens epithelial cells, leading to the separation of cell junctions and the formation of cystic vacuoles, which manifest as visible water cleft cysts [81]. The concentrations of 25(OH)D in the aqueous humor are higher in patients with diabetic cataracts compared to those with age-related cataracts [82]. Men with higher levels of serum 25-hydroxyvitamin D exhibited a reduced risk of age-related cataracts compared to those with lower levels of serum 25-hydroxyvitamin D [83]. A statistically significant association between vitamin D deficiency and the onset of early age-related cataracts was observed by Öktem Ç [84]. To sum up, the evidence linking cataract and vitamin D_3_ remains very limited and contrasting. Further extensive research is necessary to explore the connection between vitamin D deficiency and the onset of cataract.

## 12. Conclusions

While there is growing evidence supporting a potential association between vitamin D_3_ and ocular diseases such as dry eye disease, myopia, keratoconus, thyroid eye disease, retinoblastoma, age-related macular degeneration, diabetic retinopathy, glaucoma, and cataract, the available literature remains limited and sometimes contradictory. Ocular tissues contain the vitamin D receptor and its regulatory enzymes. Thus, studies have demonstrated that ocular tissues can activate and regulate vitamin D levels, emphasizing the significance of this nutrient in maintaining ocular health. As with any medical intervention, the use of vitamin D supplementation in ocular diseases should be individualized based on factors such as the patient’s overall health, vitamin D status, and response to treatment. Further, well-designed observational studies and randomized controlled trials are necessary to elucidate the precise mechanisms underlying these associations and determine whether vitamin D_3_ supplementation could be beneficial for preventing or managing ocular diseases. We suggest performing large-scale population-based studies to examine geographic variations in vitamin D levels and their correlation with the prevalence or severity of ocular diseases, taking into account factors such as sunlight exposure, dietary habits, and lifestyle factors. The exploration of the potential synergistic effects of vitamin D supplementation with other treatments commonly used for ocular diseases should be taken into consideration.

## Data Availability

All the materials and information will be available upon an e-mail request to the corresponding author.

## References

[1] Chauhan K., Shahrokhi M., Huecker M.R. (2023). Vitamin D. StatPearls.

[2] Moher D., Liberati A., Tetzlaff J., Altman D.G., PRISMA Group (2009). Preferred reporting items for systematic reviews and meta-analyses: The PRISMA statement. PLoS Med..

[3] Bhatt R.B., Patel N.H., Shah A.T., Ranpara K.H. (2023). Study of correlation between vitamin D3 levels and dry eye. Indian J. Ophthalmol..

[4] Bae S.H., Shin Y.J., Kim H.K., Hyon J.Y., Wee W.R., Park S.G. (2016). Vitamin D Supplementation for Patients with Dry Eye Syndrome Refractory to Conventional Treatment. Sci. Rep..

[5] Schwalfenberg G.K. (2011). A review of the critical role of vitamin D in the functioning of the immune system and the clinical implications of vitamin D deficiency. Mol. Nutr. Food Res..

[6] Lee V., Rekhi E., Hoh Kam J., Jeffery G. (2012). Vitamin D rejuvenates aging eyes by reducing inflammation, clearing amyloid beta and improving visual function. Neurobiol. Aging.

[7] Dikci S., Akatlı A.N., Yıldırım T. (2020). Conjunctival impression cytology and tear-film changes in cases with vitamin D deficiency. Int. Ophthalmol..

[8] Yang C.H., Albietz J., Harkin D.G., Kimlin M.G., Schmid K.L. (2017). Impact of oral vitamin D supplementation on the ocular surface in people with dry eye and/or low serum vitamin D. Contact Lens Anterior Eye.

[9] Liu J., Dong Y., Wang Y. (2020). Vitamin D deficiency is associated with dry eye syndrome: A systematic review and meta-analysis. Acta Ophthalmol..

[10] Najjaran M., Zarei-Ghanavati S., Arjmand Askari E., Eslampoor A., Ziaei M. (2023). Effect of oral vitamin D supplementation on dry eye disease patients with vitamin D deficiency. Clin. Exp. Optom..

[11] Yazar S., Hewitt A.W., Black L.J., McKnight C.M., Mountain J.A., Sherwin J.C., Oddy W.H., Coroneo M.T., Lucas R.M., Mackey D.A. (2014). Myopia is associated with lower vitamin D status in young adults. Investig. Ophthalmol. Vis. Sci..

[12] Wolf A.T., Klawe J., Liu B., Ahmad S. (2024). Association Between Serum Vitamin D Levels and Myopia in the National Health and Nutrition Examination Survey (2001–2006). Ophthalmic Epidemiol..

[13] Jiao S., Reinach P.S., Huang C., Yu L., Zhuang H., Ran H., Zhao F., Srinivasalu N., Qu J., Zhou X. (2023). Calcipotriol Attenuates Form Deprivation Myopia Through a Signaling Pathway Parallel to TGF-β2-Induced Increases in Collagen Expression. Investig. Ophthalmol. Vis. Sci..

[14] Aaraj S., Kausar A., Khan S.A. (2022). Vitamin D deficiency: A risk factor for myopia in children—A cross sectional study in a tertiary care centre. J. Pak. Med. Assoc..

[15] Li X., Lin H., Jiang L., Chen X., Chen J., Lu F. (2022). Low Serum Vitamin D Is Not Correlated with Myopia in Chinese Children and Adolescents. Front. Med..

[16] Pannu A., Vichare N., Pushkar K., Kumar A., Gupta S. (2023). Parallelism between hypovitaminosis D3 and recently detected myopia in children with amplified screen use in the COVID-19 era—A preliminary study. Indian J. Ophthalmol..

[17] Williams K.M., Bentham G.C., Young I.S., McGinty A., McKay G.J., Hogg R., Hammond C.J., Chakravarthy U., Rahu M., Seland J. (2017). Association Between Myopia, Ultraviolet B Radiation Exposure, Serum Vitamin D Concentrations, and Genetic Polymorphisms in Vitamin D Metabolic Pathways in a Multicountry European Study. JAMA Ophthalmol..

[18] Zarei-Ghanavati S., Yahaghi B., Hassanzadeh S., Mobarhan M.G., Hakimi H.R., Eghbali P. (2020). Serum 25-Hydroxyvitamin D, Selenium, Zinc and Copper in Patients with Keratoconus. J. Curr. Ophthalmol..

[19] Erdinest N., Wajnsztajn D., London N., Solomon A. (2023). Ocular surface inflammation and ectatic corneal disorders. Curr. Opin. Allergy Clin. Immunol..

[20] Awad E.A., Torky M.A., Bassiouny R.M., Khattab A.M., Elzehery R.R., Elhelaly R.M. (2023). Thyroid gland dysfunction and vitamin D receptor gene polymorphism in keratoconus. Eye.

[21] Kundu G., Shetty N., Shetty R., Khamar P., D’Souza S., Meda T.R., Nuijts R.M.M.A., Narasimhan R., Roy A.S. (2023). Artificial intelligence-based stratification of demographic, ocular surface high-risk factors in progression of keratoconus. Indian J. Ophthalmol..

[22] Aslan M.G., Fındık H., Okutucu M., Aydın E., Oruç Y., Arpa M., Uzun F. (2021). Serum 25-Hydroxy Vitamin D, Vitamin B12, and Folic Acid Levels in Progressive and Nonprogressive Keratoconus. Cornea.

[23] McMillan J. (2018). Spectrum of Darkness, Agent of Light: Myopia, Keratoconus, Ocular Surface Disease, and Evidence for a Profoundly Vitamin D-dependent Eye. Cureus.

[24] Lasagni Vitar R.M., Fonteyne P., Knutsson K.A., Bertuzzi F., Galli L., Rama P., Ferrari G. (2022). Vitamin D Supplementation Impacts Systemic Biomarkers of Collagen Degradation and Copper Metabolism in Patients with Keratoconus. Transl. Vis. Sci. Technol..

[25] Gupta P.C., Pathak M., Thakur B., Fogla R., Agarwal A., Ram J. (2022). Association of keratoconus with serum levels of 25-hydroxyvitamin D and antioxidant trace elements: A systematic review and meta-analysis. Indian J. Ophthalmol..

[26] Planck T., Shahida B., Malm J., Manjer J. (2018). Vitamin D in Graves Disease: Levels, Correlation with Laboratory and Clinical Parameters, and Genetics. Eur. Thyroid. J..

[27] Heisel C.J., Riddering A.L., Andrews C.A., Kahana A. (2020). Serum Vitamin D Deficiency Is an Independent Risk Factor for Thyroid Eye Disease. Ophthalmic Plast. Reconstr. Surg..

[28] Alhuzaim O.N., Aljohani N. (2014). Effect of vitamin d3 on untreated graves’ disease with vitamin d deficiency. Clin. Med. Insights Case Rep..

[29] Khamisi S., Lundqvist M., Rasmusson A.J., Engström B.E., Karlsson F.A., Ljunggren Ö. (2023). Vitamin D and bone metabolism in Graves’ disease: A prospective study. J. Endocrinol. Investig..

[30] Hu Y.X., Zheng R.D., Fan Y.F., Sun L., Hu X., Liu C. (2020). The effects of bone metabolism in different methylprednisolone pulse treatments for Graves’ ophthalmopathy. Exp. Ther. Med..

[31] Alali M., Alkulaib N.S., Alkhars A., Albadri K., Al Hassan S., Elewa M., Aldairi W., Alsaqer S.K., Al-Abdulqader R.A., Alhammad F. (2023). Thyroid eye disease in Eastern Province of Saudi Arabia: Clinical profile and correlation with vitamin D deficiency. Orbit.

[32] Zawadzka-Starczewska K., Stasiak B., Wojciechowska-Durczyńska K., Lewiński A., Stasiak M. (2022). Novel Insight into Non-Genetic Risk Factors of Graves’ Orbitopathy. Int. J. Environ. Res. Public Health.

[33] Lanzolla G., Di Matteo L., Comi S., Cosentino G., Menconi F., Maglionico M.N., Posarelli C., Figus M., Marinò M. (2023). Absence of a relationship between vitamin D and Graves’ orbitopathy. J. Endocrinol. Investig..

[34] Ishaq H., Patel B.C. (2023). Retinoblastoma. StatPearls.

[35] Jung E.M., Bunin G.R., Ganguly A., Johnson R.A., Spector L.G. (2023). The association between maternal nutrient intake during pregnancy and the risk of sporadic unilateral retinoblastoma among offspring. Cancer Epidemiol..

[36] Sabet S.J., Darjatmoko S.R., Lindstrom M.J., Albert D.M. (1999). Antineoplastic effect and toxicity of 1,25-dihydroxy-16-ene-23-yne-vitamin D3 in athymic mice with Y-79 human retinoblastoma tumors. Arch. Ophthalmol..

[37] Albert D.M., Kumar A., Strugnell S.A., Darjatmoko S.R., Lokken J.M., Lindstrom M.J., Patel S. (2004). Effectiveness of vitamin D analogues in treating large tumors and during prolonged use in murine retinoblastoma models. Arch. Ophthalmol..

[38] Damera G., Fogle H.W., Lim P., Goncharova E.A., Zhao H., Banerjee A., Tliba O., Krymskaya V.P., Panettieri R.A. (2009). Vitamin D inhibits growth of human airway smooth muscle cells through growth factor-induced phosphorylation of retinoblastoma protein and checkpoint kinase 1. Br. J. Pharmacol..

[39] Kulkarni A.D., van Ginkel P.R., Darjatmoko S.R., Lindstrom M.J., Albert D.M. (2009). Use of combination therapy with cisplatin and calcitriol in the treatment of Y-79 human retinoblastoma xenograft model. Br. J. Ophthalmol..

[40] Pérez Serena A., Martínez Betancourt D.P., González Del Valle F., Ruiz-Moreno J.M. (2022). Serum 25-hydroxy vitamin D levels in age-related macular degeneration. Int. J. Retin. Vitr..

[41] Kabataş N., Doğan A.Ş., Yılmaz M., Kabataş E.U., Biçer T., Çalışkan S., Çelikay O., Uçar F., Gürdal C. (2022). Association between age-related macular degeneration and 25(OH) vitamin D levels in the Turkish population. Arq. Bras. Oftalmol..

[42] Seyyar S.A., Tokuc E.O., Tıskaoğlu N.S., Karabaş V.L., Güngör K. (2022). Do serum vitamin D levels correlate with Macular Edema or with Diabetic Retinopathy?. Eur. J. Ophthalmol..

[43] Ekinci C., Guler E.M., Kocyigit A., Kirik F., Ozdemir H. (2021). Effects of 1,25 Dihydroxyvitamin D3 on Human Retinal Pigment Epithelial Cell Lines. Int. Ophthalmol..

[44] Fu Y., Chen X., Luo S., Jiang S., Mao Y., Xiao W. (2023). Serum 25-Hydroxyvitamin D Is Differentially Associated with Early and Late Age-Related Macular Degeneration in the United States Population. Nutrients.

[45] Ferreira A., Silva N., Furtado M.J., Carneiro Â., Lume M., Andrade J.P. (2021). Serum vitamin D and age-related macular degeneration: Systematic review and meta-analysis. Surv. Ophthalmol..

[46] Kan E., Kan E.K., Yücel Ö.E. (2020). The Possible Link Between Vitamin D Levels and Exudative Age-related Macular Degeneration. Oman Med. J..

[47] Lazzara F., Conti F., Platania C.B.M., Eandi C.M., Drago F., Bucolo C. (2021). Effects of Vitamin D3 and Meso-Zeaxanthin on Human Retinal Pigmented Epithelial Cells in Three Integrated in vitro Paradigms of Age-Related Macular Degeneration. Front. Pharmacol..

[48] D’Aloisio R., Di Antonio L., Toto L., Rispoli M., Di Iorio A., Delvecchio G., Mastropasqua R. (2022). Choroidal Changes in Blood Flow in Patients with Intermediate AMD after Oral Dietary Supplement Based on Astaxanthin, Bromelain, Vitamin D3, Folic Acid, Lutein, and Antioxidants. Medicina.

[49] Hernandez M., Recalde S., González-Zamora J., Bilbao-Malavé V., Sáenz de Viteri M., Bezunartea J., Moreno-Orduña M., Belza I., Barrio-Barrio J., Fernandez-Robredo P. (2021). Anti-Inflammatory and Anti-Oxidative Synergistic Effect of Vitamin D and Nutritional Complex on Retinal Pigment Epithelial and Endothelial Cell Lines against Age-Related Macular Degeneration. Nutrients.

[50] Merle B.M.J., Silver R.E., Rosner B., Seddon J.M. (2017). Associations Between Vitamin D Intake and Progression to Incident Advanced Age-Related Macular Degeneration. Investig. Ophthalmol. Vis. Sci..

[51] Aoki A., Inoue M., Nguyen E., Obata R., Kadonosono K., Shinkai S., Hashimoto H., Sasaki S., Yanagi Y. (2016). Dietary n-3 Fatty Acid, α-Tocopherol, Zinc, vitamin D, vitamin C, and β-carotene are Associated with Age-Related Macular Degeneration in Japan. Sci. Rep..

[52] Gverović Antunica A., Znaor L., Ivanković M., Puzović V., Marković I., Kaštelan S. (2023). Vitamin D and Diabetic Retinopathy. Int. J. Mol. Sci..

[53] Zahedi M., Motahari M.M., Fakhri F., Aphshari N.M., Poursharif S., Jahed R., Nikpayam O. (2024). Is vitamin D deficiency associated with retinopathy in type 2 diabetes mellitus? A case-control study. Clin. Nutr. ESPEN.

[54] Alcubierre N., Valls J., Rubinat E., Cao G., Esquerda A., Traveset A., Granado-Casas M., Jurjo C., Mauricio D. (2015). Vitamin D Deficiency Is Associated with the Presence and Severity of Diabetic Retinopathy in Type 2 Diabetes Mellitus. J. Diabetes Res..

[55] Zhuang Y., Zhuang Z., Cai Q., Hu X., Huang H. (2024). Serum vitamin D is substantially reduced and predicts flares in diabetic retinopathy patients. J. Diabetes Investig..

[56] Navaei S., Nazemi S., Emamian M.H., Hashemi H., Fotouhi A. (2023). Vitamin D deficiency and diabetic retinopathy risk. J. Fr. Ophtalmol..

[57] Maghbooli Z., Ebrahimi Meimand S., Malek Hosseini A.A., Shirvani A. (2022). Alterations in circulating levels of vitamin D binding protein, total and bioavailability of vitamin D in diabetic retinopathy patients. BMC Endocr. Disord..

[58] Hardatt D., Devi M., Vyas S., Singh G., Jain J., Gupta S., Dhanawat M. (2023). Effect of Vitamin D on Retinoblastoma Protein in Prediabetic Individuals. Curr. Diabetes Rev..

[59] Lazzara F., Longo A.M., Giurdanella G., Lupo G., Platania C.B.M., Rossi S., Drago F., Anfuso C.D., Bucolo C. (2022). Vitamin D3 preserves blood retinal barrier integrity in an in vitro model of diabetic retinopathy. Front. Pharmacol..

[60] Lu L., Lu Q., Chen W., Li J., Li C., Zheng Z. (2018). Vitamin D3 Protects against Diabetic Retinopathy by Inhibiting High-Glucose-Induced Activation of the ROS/TXNIP/NLRP3 Inflammasome Pathway. J. Diabetes Res..

[61] Lu L., Zou G., Chen L., Lu Q., Wu M., Li C. (2021). Elevated NLRP3 Inflammasome Levels Correlate with Vitamin D in the Vitreous of Proliferative Diabetic Retinopathy. Front. Med..

[62] Filippelli M., Campagna G., Vito P., Zotti T., Ventre L., Rinaldi M., Bartollino S., dell’Omo R., Costagliola C. (2021). Anti-inflammatory Effect of Curcumin, Homotaurine, and Vitamin D3 on Human Vitreous in Patients with Diabetic Retinopathy. Front. Neurol..

[63] Huang C., Luo D., Sun M., Fang G., Wei M., Zhang Y., Wang J., Huang Y. (2024). No causal association between serum vitamin D levels and diabetes retinopathy: A Mendelian randomization analysis. Nutr. Metab. Cardiovasc. Dis..

[64] Seyyar S.A., Tıskaoğlu N.S., Onder Tokuc E., Mercanlı M., Doğan L. (2023). Is serum vitamin D associated with diabetic retinopathy and its severity or with diabetes itself?. Clin. Exp. Optom..

[65] Fekri S., Soheilian M., Roozdar S., Abtahi S.H., Nouri H. (2022). The effect of vitamin D supplementation on the outcome of treatment with bevacizumab in diabetic macular edema: A randomized clinical trial. Int. Ophthalmol..

[66] Vergroesen J.E., de Crom T.O.E., Blekkenhorst L.C., Klaver C.C.W., Voortman T., Ramdas W.D. (2022). Dietary Nitrate Intake Is Associated with Decreased Incidence of Open-Angle Glaucoma: The Rotterdam Study. Nutrients.

[67] Krefting E.A., Jorde R., Christoffersen T., Grimnes G. (2014). Vitamin D and intraocular pressure--results from a case-control and an intervention study. Acta Ophthalmol..

[68] Lv Y., Yao Q., Ma W., Liu H., Ji J., Li X. (2016). Associations of vitamin D deficiency and vitamin D receptor (Cdx-2, Fok I, Bsm I and Taq I) polymorphisms with the risk of primary open-angle glaucoma. BMC Ophthalmol..

[69] Lazzara F., Amato R., Platania C.B.M., Conti F., Chou T.H., Porciatti V., Drago F., Bucolo C. (2021). 1α,25-dihydroxyvitamin D3 protects retinal ganglion cells in glaucomatous mice. J. Neuroinflamm..

[70] Lv Y., Han X., Yao Q., Zhang K., Zheng L., Hong W., Xing X. (2019). 1α,25-dihydroxyvitamin D3 attenuates oxidative stress-induced damage in human trabecular meshwork cells by inhibiting TGFβ-SMAD3-VDR pathway. Biochem. Biophys. Res. Commun..

[71] Lee J.H., Kwon Y.J., Lee H.S., Han J.H., Joung B., Kim S.J. (2023). Inverse Relationship between Serum 25-Hydroxyvitamin D and Elevated Intraocular Pressure. Nutrients.

[72] Abass I.A., Saleh A.T., Badi A.D., Mohammad B.I., Hamied F.M., Al-Aubaidy H.A. (2023). Correlation of serum 1,25-dihydroxycholecalciferol with the incidence of primary open-angle glaucoma: A cross-sectional study on patients with chronic illnesses. Saudi J. Ophthalmol..

[73] Lee T., Jammal A.A., Medeiros F.A. (2022). Association Between Serum Vitamin D Level and Rates of Structural and Functional Glaucomatous Progression. J. Glaucoma.

[74] Bokhary K.A., Alqahtani L.Y., Aljaser F.S., Abudawood M., Almubarak F., Algowaifly S., Jamous K.F., Fahmy R. (2022). Association of Vitamin D deficiency with primary glaucoma among Saudi population—A pilot study. Saudi J. Ophthalmol..

[75] Jia S., Chen F., Wang H., Kesavamoorthy G., Lai J.S., Wong I.Y., Chiu K., Chan J.C. (2021). Effect of Vitamin D3 on Regulating Human Tenon’s Fibroblasts Activity. Transl. Vis. Sci. Technol..

[76] Cho Y., Yun S.P., Yoo W.S., Kim R.B., Cho M.C., Kim S.J. (2021). Reduced 25-hydroxyvitamin D concentration in the aqueous humor of cataract patients with open-angle glaucoma. Sci. Rep..

[77] Molinari C., Ruga S., Farghali M., Galla R., Fernandez-Godino R., Clemente N., Uberti F. (2021). Effects of a New Combination of Natural Extracts on Glaucoma-Related Retinal Degeneration. Foods.

[78] Rahman S.T., Waterhouse M., Romero B.D., Baxter C., English D., Mackey D.A., Ebeling P.R., Armstrong B.K., McLeod D.S.A., Hartel G. (2023). Vitamin D Supplementation and the Incidence of Cataract Surgery in Older Australian Adults. Ophthalmology.

[79] Abdellah M.M., Mohamed Mostafa E., Salama E.H., Roshdy Mohamed E. (2019). Association of Serum 25-Hydroxyl Vitamin D Deficiency and Age-Related Cataract: A Case-Control Study. J. Ophthalmol..

[80] Liu H., Shen X., Yu T., Wang Y., Cai S., Jiang X., Cai X. (2022). A putative causality of vitamin D in common diseases: A mendelian randomization study. Front. Nutr..

[81] Brown C.J., Akaichi F. (2015). Vitamin D deficiency and posterior subcapsular cataract. Clin. Ophthalmol..

[82] Cho M.C., Kim R.B., Ahn J.Y., Yoo W.S., Kim S.J. (2020). Aqueous humor and serum 25-Hydroxyvitamin D levels in patients with cataracts. BMC Ophthalmol..

[83] Jee D., Kim E.C. (2015). Association between serum 25-hydroxyvitamin D levels and age-related cataracts. J. Cataract. Refract. Surg..

[84] Öktem Ç., Aslan F. (2021). Vitamin D Levels in Young Adult Cataract Patients: A Case-Control Study. Ophthalmic Res..

